# The Direct Involvement of Dark-Induced Tic55 Protein in Chlorophyll Catabolism and Its Indirect Role in the MYB108-NAC Signaling Pathway during Leaf Senescence in *Arabidopsis thaliana*

**DOI:** 10.3390/ijms19071854

**Published:** 2018-06-23

**Authors:** Ming-Lun Chou, Wan-Yu Liao, Wan-Chen Wei, Althea Yi-Shan Li, Ching-Ying Chu, Chia-Ling Wu, Chun-Lin Liu, Ting-Han Fu, Lee-Fong Lin

**Affiliations:** 1 Department of Life Sciences, Tzu Chi University, Hualien 97004, Taiwan; mlchou1015@gms.tcu.edu.tw (M.-L.C.); 103711117@gms.tcu.edu.tw (A.Y.-S.L.); 104711126@gms.tcu.edu.tw (C.-Y.C.); 106726102@gms.tcu.edu.tw (C.-L.W.); 2 Institute of Medical Sciences, Tzu Chi University, Hualien 97004, Taiwan; 98751101@gms.tcu.edu.tw; 3 Department of Surgery, Cheng-Ching Hospital, Chung-Kang Branch, Taichung 40764, Taiwan; wcwei0125@gmail.com; 4 Department of Molecular Biology and Human Genetics, Tzu Chi University, Hualien 97004, Taiwan; kohiyodesuyama@gmail.com; 5 Department of Medical Informatics, Tzu Chi University, Hualien 97004, Taiwan; tinghan117@gmail.com

**Keywords:** Tic55 proteins of chloroplasts, dark-induced leaf senescence, MYB108, ANAC proteins

## Abstract

The chloroplast relies on proteins encoded in the nucleus, synthesized in the cytosol and subsequently transported into chloroplast through the protein complexes Toc and Tic (Translocon at the outer/inner membrane of chloroplasts). A Tic complex member, Tic55, contains a redox-related motif essential for protein import into chloroplasts in peas. However, Tic55 is not crucial for protein import in *Arabidopsis*. Here, a *tic55-II*-knockout mutant of *Arabidopsis thaliana* was characterized for Tic55 localization, its relationship with other translocon proteins, and its association with plant leaf senescence when compared to the wild type. Individually darkened leaves (IDLs) obtained through dark-induced leaf senescence were used to demonstrate chlorophyll breakdown and its relationship with plant senescence in the *tic55-II*-knockout mutant. The IDLs of the *tic55-II*-knockout mutant contained higher chlorophyll concentrations than those of the wild type. Our microarray analysis of IDLs during leaf senescence identified seven senescence-associated genes (SAGs) that were downregulated in the *tic55-II*-knockout mutant: *ASP3*, *APG7*, *DIN2*, *DIN11*, *SAG12*, *SAG13*, and *YLS9*. Real-time quantitative PCR confirmed the reliability of microarray analysis by showing the same expression patterns with those of the microarray data. Thus, Tic55 functions in dark-induced aging in *A. thaliana* by indirectly regulating downstream SAGs expression. In addition, the expression of four NAC genes, including *ANAC003*, *ANAC010*, *ANAC042*, and *ANAC075* of IDL treated *tic55-II*-knockout mutant appeared to be downregulated. Yeast one hybrid assay revealed that only *ANAC003* promoter region can be bound by MYB108, suggesting that a MYB-NAC regulatory network is involved in dark-stressed senescence.

## 1. Introduction

The chloroplast is mainly composed of proteins encoded by the nuclear genome and synthesized as precursor proteins (preproteins) in the cytosol. During the translocation of precursor proteins, several subcomponents of the complex function as the channel/motor complex components Tic110, Tic40, cpHsp70, Hsp90C, and Hsp93/ClpC; redox-regulatory subunits, Tic62, Tic55, and Tic32; and an alternative import channel Tic20/Tic21, Tic22, Tic214, Tic100, and Tic56 [1,2,3,4,5,6,7,8,9]. The transit peptide of a preprotein is further translocated across the inner membrane through the Tic20/Tic21 channel dependently or independently of Tic110 and translocate across the inner membrane [9,10,11,12]. The transit peptide is subsequently cleaved by the stromal processing peptidase (SPP) and the processed mature protein is pulled into the chloroplast’s stroma.

In the chloroplasts, photosynthesis relies on the redox reaction. Tic110 itself contains one or two regulatory disulfide bridges [13]. The formation/dissolving of such intramolecular bonds within Tic110 can influence the structure and function of this central Tic component. In addition to the redox state of the thiol system, the NADP^+^/NADPH ratio is a direct measurement of the total stromal redox state. Hirohashi and others [14] demonstrated that the redox state within the chloroplasts may regulate protein targeting. Stengel and others [15] recently demonstrated that the redox state regulates protein import into the chloroplasts and mitochondria. Three redox proteins, Tic55, Tic62, and Tic32, are associated with Tic110; among these, Tic62 can relay the redox status of the stroma to Tic110 [16]. Similar to Tic62, Tic32 dissociates from Tic110 in the presence of NAPDH, suggesting that Tic32 can also be part of a redox-signal transducer [17]. Moreover, Tic55 is associated with Tic62 and Tic32 and contains a redox-related motif essential for protein import into the chloroplasts in peas [8,18,19,20]. However, redox-motif of Tic55 is essential for its biological function in *Arabidopsis* remains unclear.

Previous studies showed Tic55 may not be involved in the protein import into chloroplasts like other translocon proteins in *Arabidopsis thaliana* and rather functions as a hydroxylase of phyllobilins during plant senescence [21,22]. Senescence in green plants is a highly controlled and complicated process. Chlorophyll degradation is the first visible sign of senescence. Arabidopsis and other higher organisms contain four classes of tetrapyrroles and chlorophyll is the most abundant tetrapyrroles that function as photosynetic pigments to harvest light energy and transfer the absorbed energy to the reaction center for the photosynthetic reactions to occur. Leaf yellowing, caused by chlorophyll degradation, is the most apparent indication of senescent leaves. The role of phyllobilin modification, mediated by the hydroxylase activity of Tic55, during chlorophyll breakdown is still not clear [22]. Interestingly, chlorophyll degradation can be resulted from a numerous occasions of endogenous and environmental cues. The environmental factors that influence leaf senescence include abiotic and biotic factors. The abiotic influence is attributed to the drought, nutrient limitation, extreme temperature, and oxidative stress, etc. Pathogen infection, on the other hand, is one kind of biotic factors. Nevertheless, leaf senescence can occur prematurely under such unfavorable environmental stresses [23,24]. Thus, the net loss of chlorophyll in chloroplasts is the main cause of phenotypic change of senescing leaves from green to yellowing. Several lines of evidence have identified dozens of senescence-related mutants and hundreds of senescence-associated genes (*SAGs*) implicated in hormone signaling, chlorophyll catabolism, and light signaling [25,26,27]. For example, the famous stay-green pea used by Mendel has been shown to be due to the mutation in the gene encoding pheophorbide *a* oxygenase [28]. In these mutants, only the chlorophyll degradation pathway is affected. Mutants with single gene mutation usually do not lead to blocking of all aspects of senescence. There are multiple signaling pathways involved in the gene expression during senescence, and it is very likely that a single gene does not control all the signaling pathways implicated in senescence. In an experiment comparing gene expression levels at three stages of leaf development (MG (mature green); S1 (early senescence, no chlorosis) and S2 (mid senescence, 5–15% chlorosis)), Buchanan-Wollaston and coworkers [29] have identified more than 1400 genes that showed relative changes in expression during leaf development. Although some gene expression may be specific to certain stress stimulation, there is often a large overlap among different stress responses. This notion suggests that common features are possibly involved [30].

The main purpose of plant senescence is to mobilize and recycle. When the leaf is no longer needed by the plant, the senescence process is triggered to relocate all the nutrients from leaves to reproducing seeds or to other growing organs. During this process, plant-specific NAC (NAM/ATAF1, 2/CUC2) family of transcription factors (TFs) in controlling the stress responses may play vital roles in plant senescence [31,32]. NAC TFs by far is the largest plant TF families with over 100 members in *Arabidopsis* for instance [33]. NAC TFs have been related to a variety of stress-associated responses such as drought, high salinity, bacterial and fungal pathogens, and senescence [24,34,35,36,37,38]. Many members of NAC family appear to have overlapping expression patterns and are involved in regulating multiple stress responses, a situation suggesting their common roles in regulation [24,38,39]. From the recent structural and functional studies, NAC TFs proteins contain a highly conserved target-specific N-terminal DNA binding domain and a divergent C-terminal domain, which interacts dynamically with other proteins and functions as a transcription regulatory domain (TRD) [40,41,42,43]. NAC TFs interact and control other NACs or other TFs to fine-tune the target genes expression, thereby forming a NAC regulatory network to integrate multiple developmental and environmental signals [44,45]. Within this network, NAC, WRKY, and MYB TFs are central key players in regulating transcriptional changes during senescence. Therefore, it appears that leaf senescence is controlled through multiple and cross-linking pathways, many of which are related to stress response signaling [32,38,45,46,47].

In this study, in order to address whether the redox status of Tic55 in *Arabidopsis* affects its biological function, a *tic55-II*-knockout mutant line of *Arabidopsis thaliana,* SALK_086048, randomly chosen and obtained from the Arabidopsis Biological Resource Center (ABRC), was characterized. The *tic55-II*-knockout mutant revealed no significant physical differences, and the change in the redox state did not affect the association of this mutant with other translocon proteins. This indicates that Tic55 does not have a crucial role in *A. thaliana* survival and implies that Tic55 may not function as a redox signal transducer implicated in its biological activities. By contrast, dark-induced leaf senescence experiments (IDLs) [48] have revealed a unique biological function of Tic55 as an aging-related protein in *A. thaliana*. Chlorophyll concentrations were higher in the *tic55-II*-knockout mutant than in the wild type (WT). Microarray gene expression analysis of individually darkened leaves (IDLs) during the leaf senescence of *A. thaliana* revealed 830 transcripts assigned to three main gene ontology (GO) categories, namely biological processes, molecular functions, and cellular components, and 111 subcategories based on TAIR (http://www.arabidopsis.org/help/helppages/go_slim_help.jsp) [49,50]. Seven downregulated senescence-related genes—*ASP3* (*aspartate aminotransferase 3*), *APG7* (*autophagy-related 7*, ubiquitin-like modifier-activating enzyme ATG7), *DIN2* (*dark inducible 2*, beta-glucosidase 30), *DIN11* (*dark inducible 11*, 2-oxoacid-dependent dioxygenase-like protein), *SAG12* (*senescence-associated gene 12*, cysteine protease), *SAG13* (*senescence-associated gene 13*, senescence-associated protein), and *YLS9* (*yellow-leaf-specific gene 9*, protein NDR1/HIN1-like 10)—were selected for further analysis [51,52,53,54,55,56,57,58]. Real-time quantitative reverse-transcription PCR (qRT-PCR) of these seven genes in the Columbia WT and *tic55-II*-knockout mutant after IDL treatment indicated the reliability of the microarray results. Thus, these seven senescence-associated genes (*SAGs*), including *SAG12* and *SAG13* shown previously [52,53,57] as well as in our study, are indeed involved in plant senescence. We also identified from our microarray data four NAC containing proteins, including ANAC003, ANAC010, ANAC042, and ANAC075, whose expressions were downregulated in dark-induced aging in the *tic55-II*-knockout mutant. Earlier studies have shown that the downstream gene expression of *SAGs* is possibly associated with the upstream MYB-NAC transcription factors (TFs) controlling signaling pathways [32,59]. The key mechanism of regulation in differential gene expression is that TFs function through sequence-specific binding to the promoters of target genes. Our yeast one-hybrid data revealed that MYB108 indeed interacts with the promoter of *ANAC003*, indicating MYB-NAC direct regulatory roles in dark-induced senescence in the *tic55-II*-knockout mutant. We present not only the novel biological function of Tic55 in dark-induced aging in *A. thaliana*, but also this stress-specific regulatory pathway.

## 2. Results and Discussion

### 2.1. Molecular Characterization and Phenotype of T-DNA Insertion Mutant tic55-II

**Figure 1:**
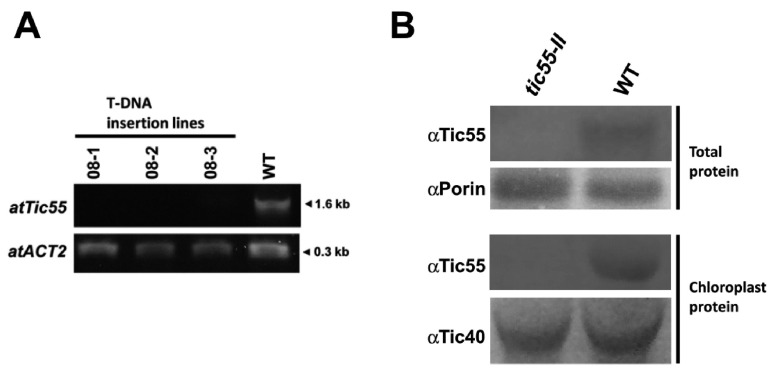
Molecular characterization of T-DNA insertion lines derived from SALK_086048. (**A**) RT-PCR analysis was performed using RT-F and RT-R primers as indicated in Table S1. *Tic55* in *A. thaliana* is shown as *atTic55*. Internal control was *atACT2*, an actin gene in *A. thaliana*, used to normalize sample loading. (**B**) Protein gel blotting assay for both total protein and chloroplast protein was performed to detect Tic55 in the wild-type (WT) and knockout mutant (*tic55-II*) plant extracts by using a Tic55-specific antibody (αTic55) or the housekeeping protein porin (αPorin) and Tic40 (αTic40).

**Figure 2:**
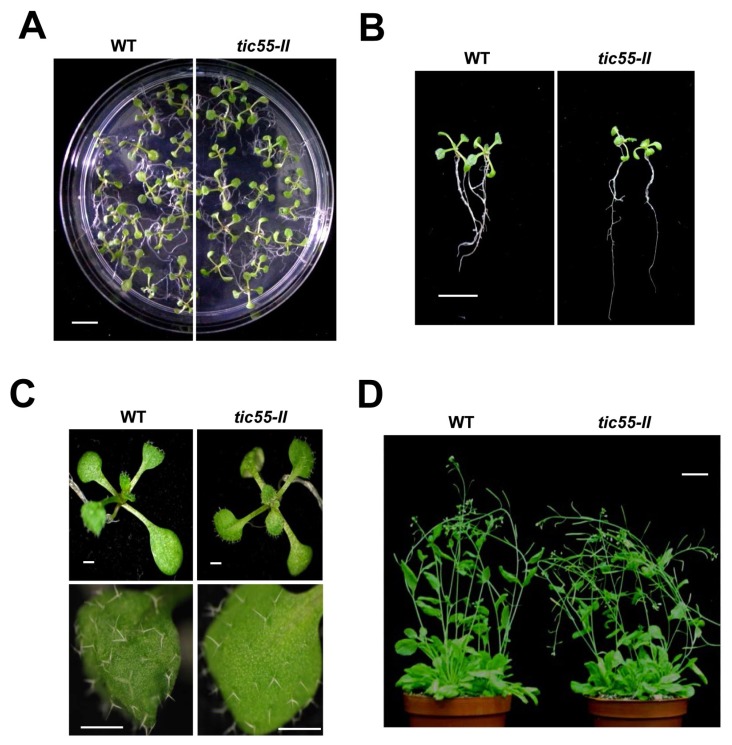
Phenotypes of the wild-type (WT) and *tic55-II*-knockout mutant lines. (**A**) WT and *tic55-II*-knockout (SALK_086048) mutant lines were grown on MS medium side by side for 10 days, and the appearance of seedlings were compared. White bar indicates 1 cm. (**B**) Comparison of root systems of 10-day-old WT and *tic55-II*-knockout mutant seedlings, respectively. White bar represents 1 cm. (**C**) Leaf tissues in (**A**) were photographed at closer distances for both WT and *tic55-II*-knockout mutant plants (upper panel). Lower panel shows the number of leaf hairs for both WT and *tic55-II*-knockout mutant plants. White bar (white line) indicates 1 mm. (**D**) Similar plants were grown in vitro for 10 days and then transferred to soil and grown for 30 days. White bar represents 2 cm.

To assess the biological importance of Tic55 in *A. thaliana*, *tic55* knockout mutant line with T-DNA insertion in *tic55* (AT2G24820), named SALK_086048, was obtained from the Arabidopsis Biological Resource Center (ABRC). Both the WT and *tic55* knockout mutant plants were grown on Murashige and Skoog (MS) agar with kanamycin, and screening revealed the presence of T-DNA insertion. The T-DNA insertion site in the mutant line was confirmed through genomic PCR, followed by DNA sequencing at the T-DNA/gene junctions. In this knockout mutant, T-DNA disrupted the first exon of *Tic55*. These mutant lines were further confirmed to be true *tic55* knockout plants through RT-PCR and protein gel blot analysis. Although no mutant (08-1, 08-2, or 08-3) showed the presence of the 1.6-kb full-length transcript, it was noted in the WT (Figure 1A). Protein gel blot analysis with a Tic55-specific antibody (αTic55) further confirmed the complete absence of Tic55 in the total protein and chloroplast of the knockout line, named *tic55-II* (Figure 1B). These data corroborate those of Boij et al. [21], who screened and characterized three *tic55* knockout mutants, including SALK_086048. We therefore obtained a true *tic55* knockout mutant in order to further study the potential biological functions of Tic55 in *A. thaliana*. Both WT and *tic55-II*-knockout plants were grown on MS agar and soil to observe abnormal phenotypes. No significant phenotypic differences were observed (Figure 2).

### 2.2. The Relationship between Tic55 and Other Translocon Proteins

Most chloroplast proteins are nuclear-encoded preproteins that are synthesized in the cytosol, directed to the chloroplast by the transit peptide at the N-terminus of the preprotein, and translocated into the chloroplast. After successful import, the transit peptide is cleaved off by the SPP, resulting in the mature form of the protein. Eight proteins are involved in preprotein import at the IM of the chloroplast: Tic110, Tic62, Tic55, Tic40, Tic32, Tic22, Tic21, and Tic20 [4]. Recently, Tic56, Tic100, and Tic214 were reported to be associated with Tic20 to form the 1 MDa complex at the IM, which also functions as a translocation channel for preproteins [9,12]. In addition, a defect in Tic110 resulted in the death of mutant plants, indicating that Tic110 is essential in protein import machinery [60]. However, the *tic55-II*-knockout mutant plants exhibited no significant phenotypic differences from the WT (Figure 2), indicating that Tic55 may not be functionally critical to the survival of *A. thaliana* in normal growth conditions.

**Figure 3:**
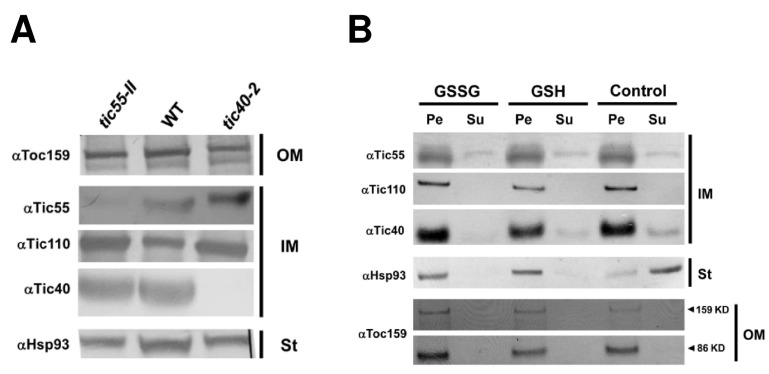
Relationship of Tic55 and other translocon proteins. (**A**) Protein gel blot analysis demonstrating the location of the inner membrane proteins Tic110 and Tic40, the outer membrane protein Toc159, and the stoma protein Hsp93 in *tic55-II*-knockout or *tic40-2* mutants and WT plants. (**B**) Location of Tic55 and other translocon proteins under redox environments. Chloroplast fraction was treated with oxidized glutathione (GSSG) and reduced glutathione (GSH). Control indicates no treatment. After ultracentrifugation, lipid-soluble (Pe) and water-soluble (Su) protein fractions were separated and examined using protein gel blots to explore the location and complex formation of Tic55 with other translocon proteins. IM: inner membrane. St: stroma. OM: outer membrane.

Boij and others [21] demonstrated that only atTic55-II, but not AtPTC52 (atTic55-IV), can be considered a plausible ortholog of the pea Tic55 protein (psTic55) because the amino acid sequences of atTic55 and AtPTC52 are only 26% identical, but they are 79% identical for psTic55 and atTic55-II. To determine whether atTic55-II would affect the level of translocon proteins involved in the protein import machinery, total proteins from 14-day-old WT, *tic55-II*-knockout, and *tic40-2* mutant lines under normal growth conditions were extracted. Between the *tic55-II*-knockout mutant and WT, protein quantities were similar for the outer membrane component Toc159, the inner membrane components Tic110 and Tic40, and the stromal protein Hsp93 (Figure 3A). Therefore, a defect in Tic55 does not affect the cellular levels of other translocon proteins essential to precursor protein translocation into the chloroplast stroma.

Tic55 is located in the inner membrane of chloroplast in peas [19]. To determine the location of Tic55 in *A. thaliana*, chloroplasts were isolated through fractionation analysis and separated into four parts: the IM, OM, thylakoid membrane, and stroma. Protein gel blots indicate that Tic55 was fractionated in the IM of chloroplast in *A. thaliana* (Figure S1A and Figure 3A).

**Figure 4:**
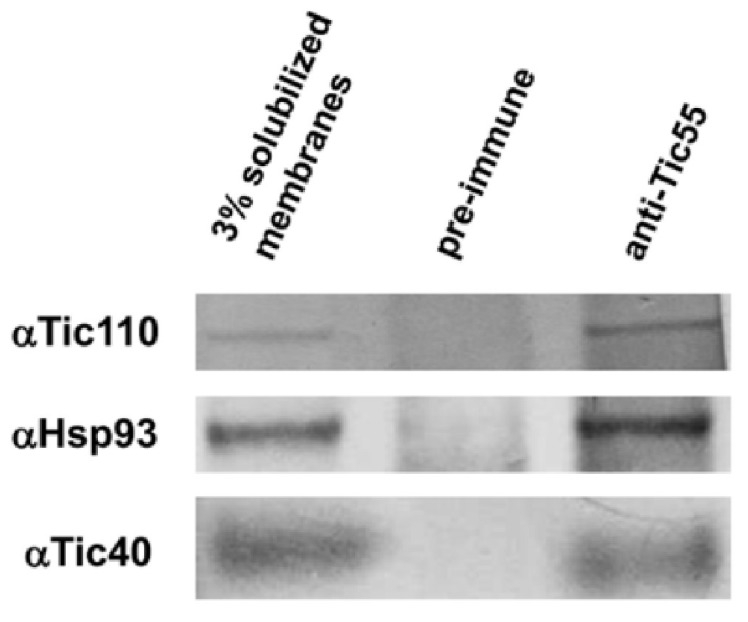
Tic55 could directly interact with other Tic proteins. Coimmunoprecipitation (Co-IP) analysis was used to determine complex formation between Tic55 and other translocon proteins. Isolated chloroplasts from wild-type *A. thaliana* were treated with ice for 15 min, followed by hypotonic treatment. Chloroplast membrane proteins were subsequently retrieved, and an immunoprecipitation (IP) assay was performed using either anti-Tic55 antibody or preimmune serum (negative control). Next, protein gel blots were conducted using specific antibodies αTic110, αHsp93, or αTic40 to detect whether a particular translocon protein could be coimmunoprecipitaed to indicate that the protein can form a complex with Tic55 protein.

Furthermore, reports have demonstrated that in *A. thaliana*, AtTic55 (At2g24820) has a role in redox regulation and is possibly regulated by its thioredoxins [19,61]. Other translocon proteins alter protein-protein interactions depending on the cellular redox state. For example, Tic62 and Tic32 form distinct complexes with nearby proteins under oxidizing conditions (high NADP^+^/NADPH ratio) compared with those under reducing conditions (low NADP^+^/NADPH ratio) [62]. Similarly, to explore whether the redox environment affects the complex formation of Tic55 with other translocon proteins, we first confirmed whether Tic55 forms a complex with other translocon proteins, including Tic110, Tic40, and Hsp93, through coimmunoprecipitation (CO-IP) assays. Isolated chloroplast membrane proteins were immunoprecipitated with either specific anti-Tic55 antibody or preimmune serum (negative control), followed by protein gel blot assay with antibodies against Tic110, Tic40, or Hsp93. Tic55 appeared to form a complex with Tic110, Tic40, and Hsp93 (Figure 4). Furthermore, we used oxidized glutathione (GSSG) and reduced glutathione (GSH) to create oxidizing and reducing environmental conditions, respectively, for the isolated chloroplasts, followed by protein gel blot analysis with a specific antibody to detect particular translocon proteins. As shown in Figure 3B, Tic55 fractionated with Tic110 and Tic40 at the IM in the presence of either GSSG or GSH, indicating the redox environments did not affect the protein–protein interactions of Tic55 and its nearby translocon proteins.

### 2.3. Possible Novel Biological Function of Tic55 in the Aging of A. thaliana

**Figure 5:**
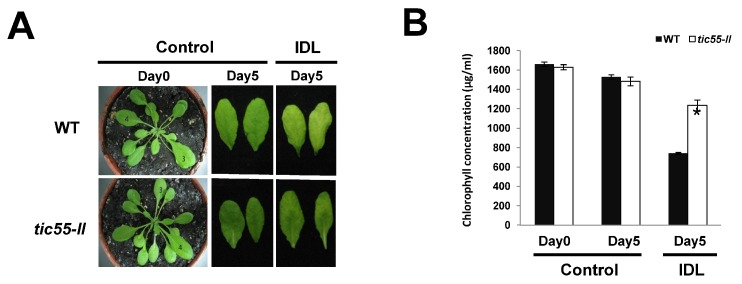
Dark-induced aging and the importance of Tic55 in senescence. (**A**) Phenotypes of wild-type (WT) and *tic55-II*-knockout mutant lines without treatment on both Day 0 and Day 5 (Control Day 0 and Control Day 5, respectively) compared with the IDLs of WT and *tic55-II*-knockout mutant lines at Day 5 after dark treatment (IDL Day 5). The third and fourth rosette leaves of Day 0 plants were individually covered with foil soon after bolting, and the plants were grown for another five days. (**B**) Quantitative chlorophyll analysis: The fifth and sixth leaves of 19-day-old seedlings were collected as Day 0 samples. After five days, the fifth and sixth leaves of the control seedlings (without dark treatment) were gathered as Day 5 samples. The fifth and sixth leaves of dark-treated plants were collected five days after treatment started on Day 0 (IDL Day 5). Chlorophyll was extracted and quantified (µg/mL) in leaves gathered from each group. Solid bars represent WT and open bars indicate *tic55-II*-knockout mutant lines. Three independent experiments were performed and standard deviation was measured. Asterisk (*) depicts *p* < 0.05.

Our data indicate that the physical appearance, other translocon proteins levels, and Tic complex formation under oxidizing or reducing environments do not differ between the *tic55-II*-knockout mutant and the WT. Dark-stimulated aging has a major effect on chlorophyll degradation in plants. Notably, similar to Tic55, some proteins from the LLS1-related nonheme family are involved in different stages of chlorophyll metabolism. For example, PAO and CAO are implicated in chlorophyll degradation [63]. Studies have also shown that LLS1 suppressed cell death in maize cells [64]. Therefore, Tic55 may play a role in the aging of *Arabidopsis* [65]. To test this hypothesis, dark-induced leaf senescence experiments (IDLs) [48] were conducted on soil-grown WT and *tic55-II*-knockout mutant plants. Dark treatment was applied to the expanding third and fourth rosette leaves (Figure 5A, Control Day 0) when both WT and *tic55-II*-knockout mutant plants were grown under long-day conditions. No apparent differences were noted between WT and *tic55-II*-knockout mutant plants in the control leaves (unshaded leaves) after five days of growth (Figure 5A, Control Day 5). However, IDLs of the *tic55-II*-knockout mutant exhibited a greener phenotype than did the WT (Figure 5A, Control Day 5 and IDL Day 5). We also measured the total chlorophyll concentrations of the WT and *tic55-II*-knockout mutant plants, our data showed that at Day 5, IDLs from the mutant plants retained significantly more chlorophyll than did the IDLs from the WT plants (Figure 5B). Thus, these results indicate reduced chlorophyll degradation in the dark and in the absence of Tic55, suggesting Tic55 may play a role in leaf senescence. Notably, our results corroborate the recent findings that Tic55 functions as a hydroxylase in chlorophyll breakdown during plant senescence [22].

### 2.4. Microarray Gene Expression Analysis in IDLs during Leaf Senescence of A. thaliana

**Figure 6:**
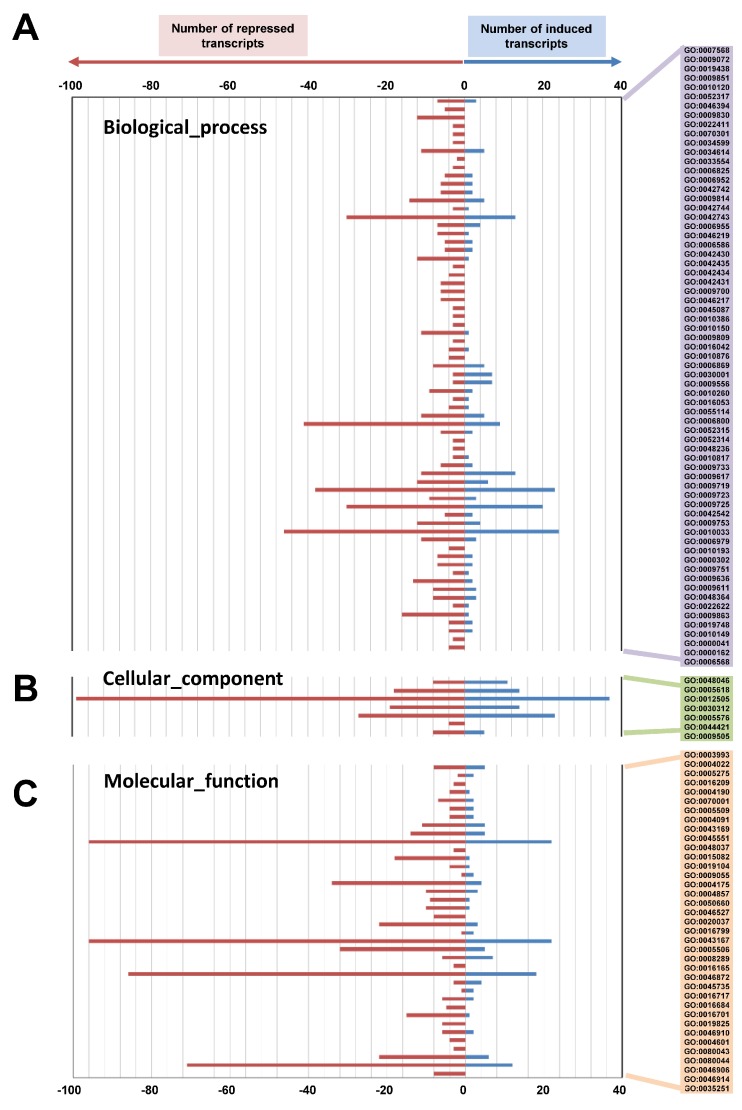
Regulated genes (log_2_-fold change) assigned to functional protein categories based on the gene ontology (GO) classification scheme. These genes are involved in the biological processes (**A**), cellular components (**B**), or molecular functions (**C**). Positive and negative values on the scale indicate the numbers of significantly up- and downregulated genes, respectively.

Dark-induced leaf senescence experiments revealed that *tic55* gene knockout led to the delay of leaf aging (Figure 5) and decrease in the expression of some senescence-associated genes (*SAGs*) in *A. thaliana*. To obtain further insight into whether a defect in Tic55 affects other biological pathways during leaf aging, we performed transcriptomic analysis by using microarray technology. Both WT and *tic55-II*-knockout mutant plants were provided dark-induced senescence treatment (IDL treatment) for three days, following which their leaves were collected. After a microarray assay, as indicated in the Materials and Methods section, differential gene expression associated with biological processes, cellular components, or molecular functions was analyzed (Figure 6). A log_2_ fold cutoff (*p* < 0.05) was applied to the data, resulting in a total of 923 highly significant differentially expressed genes (Figure 6). On comparing the IDLs of the *tic55-II*-knockout mutant and the WT, we identified 252 induced and 671 repressed transcripts after three days of growth. The fold change expression data was then assigned to a functional category according to the GO classification system for plants developed at TAIR (http://www.arabidopsis.org/help/helppages/go_slim_help.jsp) [49,50]. The functions of the identified genes cover various biological processes, cellular components, and molecular functions. In total, 830 transcripts were grouped into three main GO categories and 111 subcategories (functional groups) (Table S2).

### 2.5. Functional Categorization of log_2_-Fold Induced and Repressed Genes in the IDLs Based on Microarray Analysis

The patterns of transcript fold changes for upregulated and downregulated genes were further examined and classified according to their GO functional categories derived from our microarray analysis (Table S2). The trend for each subcategory presented a significant increase (*p* < 0.05) in the total number of differentially regulated (repressed and induced) genes from the IDLs of *tic55-II*-knockout mutants after dark-induced leaf senescence. Of these differentially expressed transcripts, the repressed genes increased the most in each subcategory (Table S2). Thus, our microarray data indicated that dark-induced aging in the *tic55-II*-knockout mutant, which has the defective Tic55, would subsequently affect the downstream genes expression involved in regulating the developmental stages in *A. thaliana*.

### 2.6. Confirmation of Microarray Results by Real-Time qRT-PCR

The results of our dark-induced leaf senescence experiments in *tic55-II*-knockout mutant showed increased chlorophyll concentration compared to WT (Figure 5B), and differential gene expression involved in the regulation of leaf aging in our microarray data in the absence of Tic55 (Figure 6, Table S2). In addition, a developmental tissue-specific expression profile for *atTic55* revealed that the highest expression levels are in the photosynthetic tissues, according to publicly available Affymetrix GeneChip microarray data, accessed using the Genevestigator analysis tool (www.genevestigator.ethz.ch/). Notably, *Tic55-II* expression is augmented not only in the cotyledons, sepals, and cauline leaves, but also in the senescent leaves in terms of tissue-specific expression, suggesting that *Tic55-II* gene expression increases in response to leaf aging [21]. To further explore whether Tic55 is crucial in regulating senescence-associated genes (SAGs) expression, we conducted microarray analysis using dark-induced aging leaves from the *tic55-II*-knockout mutant and identified differentially expressed genes in this mutant when compared to the WT (Table S2). Our microarray data revealed that several genes are classified into the biological processes categories divided into the subcategories of “aging” (three upregulated and seven downregulated), “senescence” (four downregulated), and “leaf senescence” (four downregulated) (Table S2). We thus selected seven genes for further analysis on the basis of their possible involvement in leaf senescence in *A. thaliana* [51,52,53,54,55,56,57,58]: *ASP3*, *APG7*, *DIN2*, *DIN11*, *SAG12*, *SAG13*, and *YLS9*. According to microarray analysis, these genes are downregulated in the *tic55-II*-knockout mutant (Table 1), indicating the aging-related gene expression is repressed under dark-induced senescence in the absence of functional Tic55, thus delaying leaf senescence.

**Figure 7:**
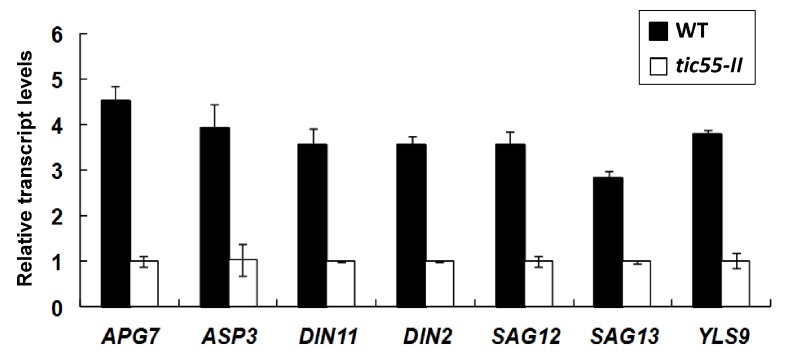
Validation of microarray expression data by relative real-time quantitative RT-PCR. Validated leaf senescence-related genes, namely *ASP3*, *APG7*, *DIN2*, *DIN11*, *SAG12*, *SAG13*, and *YLS9*, showed downregulation in the *tic55-II*-knockout mutant after dark-induced leaf senescence compared with the wild-type (WT) in microarray analysis. Relative transcript levels of these genes were determined using three replicates, and signal intensities for each transcript were normalized with tubulin (internal control). Error bars represent standard deviation. Primers used in PCR reactions are listed in Table S1. Each experiment was repeated three times with similar results. Black and white boxes indicate the Columbia WT and *tic55-II*-knockout mutant, respectively.

To confirm the differential gene expression profiles obtained from microarray assay, we validated expression values through relative real-time qRT-PCR. Both IDLs from Columbia WT and *tic55-II*-knockout mutants were collected, followed by real-time qRT-PCR. All seven genes showed the same expression patterns as they did in the microarray analysis, demonstrating the reliability of the microarray results (Figure 7). However, the fold changes determined through microarray analysis and real-time qRT-PCR were slightly different. This may have been due to technical differences in the analysis and normalization methods. Nevertheless, our results demonstrated that the expression of these aging-related genes decreased without functional Tic55 in the *tic55-II*-knockout mutant under dark treatment, thus delaying leaf senescence and causing greener phenotypes in their leaves than in those of the WT (Figure 5A). Therefore, the biological function of Tic55 is involved in plant senescence. Our data corroborate the recent reports of Hauenstein et al. [22], who demonstrated that Tic55 is involved in the hydroxylation of phyllobilins, the products of chlorophyll breakdown during senescence. These authors mainly focused on the elucidation of biological activity of Tic55, whereas our study revealed not only the direct effect of Tic55 on chlorophyll catabolism but also its indirect role in the downstream senescence associated genes (SAGs) expression through microarray and qRT-PCR analyses. Consequently, we propose that Tic55 expression is increased when a leaf enters the normal aging stage, subsequently affecting the downstream senescence-associated gene expression, leading to leaf senescence.

### 2.7. Sequence Alignment and Phylogenetic Analysis of ANAC TFs

**Figure 8:**
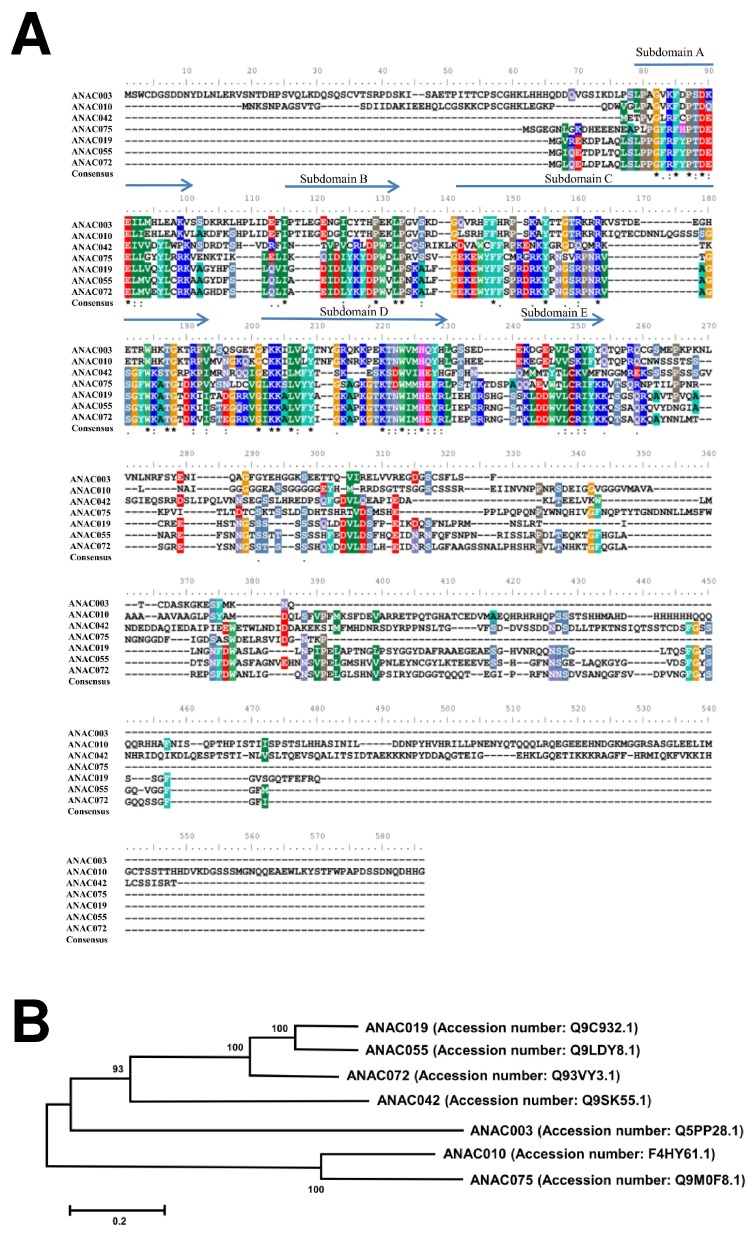
ANAC domain structure of seven *Arabidopsis* NAC transcription factors (TFs). (**A**) Architecture of NAC containing domains of seven *Arabidopsis* NAC TFs. * indicates the identical amino acids aligned within the different ANAC proteins. (**B**) phylogenetic relationships of four downregulated ANAC proteins, including ANAC003, ANAC010, ANAC042, and ANAC075 with other ANAC proteins that have been previously published involved in plant senescence.

Multiple layers of regulation are involved in the control of leaf senescence. An overview of these multiple controlling layers has been shown recently, including chromatin-mediated, transcriptional, post-transcriptional, translational, and post-translational modes of regulation [44]. From the onset through the completion of leaf senescence, it is a highly coordinated process and involves an extensive rearrangement of gene expression [44,66]. The regulation of plant senescence has been related to thousands of senescence-associated genes (*SAGs*) [52,53,57], including *SAG12* and *SAG13*, which we have found downregulated from our microarray data and confirmed by qRT-PCR analysis in the dark-induced *tic55-II*-knockout mutant, which led to delayed leaf senescence. Earlier studies have shown that *Arabidopsis SAG12* is an important molecular marker specific for leaf senescence, while not detected in the hypersensitive response (HR) linked programmed cell death (PCD) in tobacco. Similarly, *HSR2037* is upregulated during HR, but not in leaf senescence [67]. This observation indicates that signaling pathway linked leaf senescence-associated cell death is distinct from those of other HR PCDs. Thus, our results are in accord with this notion in which expression of *SAGs* is highly associated with the process of leaf senescence. Although leaf senescence is considered a complex process, upstream NAC transcription factors (TFs) appear to play essential roles in senescence and over 100 NAC genes have been found in *Arabidopsis* by transcriptome analyses, thereby implicating NAC genes as important regulators of the senescence process [33,59]. From our microarray data of dark-induced senescence in *tic55-II*-knockout mutant, we identified four downregulated genes encoding NAC domain-containing proteins, including *ANAC003*, *ANAC010*, *ANAC042*, and *ANAC075*, whereas no upregulated NAC genes were found. The common subdomains (A–E) of protein sequences were determined by MEME/MAST analysis [68] and MEGA 6.0 [69] was used to construct the phylogenetic tree by the neighbor-joining method with 1000 bootstrap replicates [70,71]. As shown in Figure 8A, alignment of four downregulated *Arabidopsis* NAC-containing proteins, ANAC003, ANAC010, ANAC042, and ANAC075, in response to dark stress in *tic55-II*-knockout mutant appeared the typical domain structure (subdomains A–E) at N-terminal end as other NAC-containing proteins, such as ANAC019 whose crystal structure was identified [41]. Our results therefore are in agreement with those of earlier studies [24,42] in that the N-terminal portion with the NAC domain of our four downregulated NAC proteins is highly conserved, whereas the C-terminal region is divergent (Figure 8A). According to the study of Podzimska-Sroka and others [72], ANAC003 and ANAC042 are senescence-associated proteins in *Arabidopsis*. Their notion further supports our microarray data in that downregulated *ANAC003* and *ANAC042* found in *tic55-II*-knockout mutant are very likely to be associated with leaf senescence. In addition, recent studies have revealed that plant signaling pathways are composed of complicated network with cross-talks [73]. In fact, several *Arabidopsis* NAC genes were identified as convergence points between different pathways, such as *ANAC019*, *ANAC055* [35,74] and *ANAC072* (*RD26)* [34], indicating the important role of NAC genes involved in plants in response to abiotic and biotic stresses. Furthermore, these three genes were found to be age-related NAC genes [32]. To analyze the relative relationship among the identified NAC proteins, phylogenetic tree of these NAC TFs and other NAC proteins was constructed as illustrated in Figure 8B.

### 2.8. MYB-NAC Linked Regulatory Pathway and Leaf Senescence

**Figure 9:**
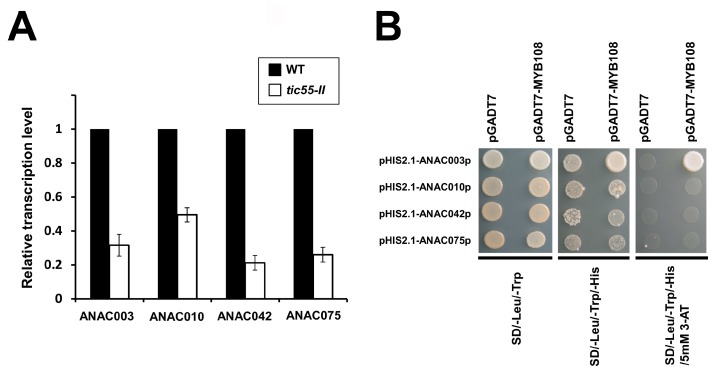
Downregulated expression of four *ANAC* genes accessed by relative real-time quantitative RT-PCR and MYB-NAC relationship determined by yeast one-hybrid assay. (**A**) Validated leaf NAC transcription factor genes, including *ANAC003*, *ANAC010*, *ANAC042*, and *ANAC075* showed downregulation in the *tic55-II*-knockout mutant after dark-induced leaf senescence compared with the wild-type (WT) in microarray analysis. Relative transcript levels of these genes were determined using three replicates, and signal intensities for each transcript were normalized with tubulin (internal control). Error bars represent standard deviation. Primers used in PCR reactions are listed in Table S1. Black and white boxes indicate the Columbia WT and *tic55-II*-knockout mutant, respectively. (**B**) Interaction between MYB108 TF and promoters of different *ANACs* was analyzed. Promoter region of different *ANAC* genes was linked to the *HIS3* reporter gene, resulting in different constructs: pHIS2.1-*ANAC003p*, pHIS2.1-*ANAC010p*, pHIS2.1-*ANAC042p* and pHIS2.1-*ANAC075p*. Each of these constructs was transformed into yeast cells either with plasmid carrying an Activation Domain (AD)-MYB108 TF fusion (pGADT7-MYB108) or pGADT7 plasmid as a negative control. Binding of MYB108 to the cis-elements of *ANAC003*, *ANA010*, *ANAC042*, or *ANAC075* promoter region, resulting in the expression of reporter gene, showed the growth of yeast cells in SD/–Leu/–Trp/–His medium in the presence of 3-amino-1,2,4-triazol (3-AT).

To validate whether NAC transcription factor genes, including *ANAC003*, *ANAC010*, *ANAC042*, and *ANAC075* are repressed in the *tic55-II*-knockout mutant after dark-induced leaf senescence, relative real-time quantitative RT-PCR assays were conducted. As shown in Figure 9A, these four NAC genes were indeed downregulated in the *tic55-II*-knockout mutant following dark-induced leaf aging when compared with the wild type (WT). Furthermore, members of a MYB TF family group, including MYB2, MYB21, MYB108, and MYB112 have been found to interact with the promoter of a NAC gene, *ANAC055*, in a sequence-specific manner [32]. In agreement with this notion is the presence of a MYB binding motif [32,75]. Thus, members of this MYB subgroup are involved in regulating the same stress responses as the NAC TFs. MYB108 revealed to be implicated in response to ABA, JA, and ethylene and also involved in regulating the response to pathogen infection [76,77]. In addition, Hickman and coworkers [32] have shown a gene regulatory network in which the expression of *ANAC019*, *ANAC055*, and *ANAC072* was regulated directly by the upstream MYB108, which was determined by using the yeast one-hybrid assays. Since we identified downregulated *MYB108* as well as four *ANAC* genes, including *ANAC003*, *ANAC010*, *ANAC042*, and *ANAC075* from Microarray analysis of dark stressed *tic55-II*-knockout mutant, similar yeast one-hybrid experiments were performed. Our data showed that indeed MYB108 interacts with the promoter of *ANAC003* gene, indicating dark-induced leaf senescence in *tic55-II*-knockout mutant is associated with the MYB108 linked NAC regulatory pathway (Figure 9B). In addition, PCR reactions confirmed that both MYB108 and NAC constructs were introduced into yeast cells and thus the results of yeast one-hybrid assays were reliable. The possible MYB TF binding sites for all four *ANAC* genes are exhibited in Table 2. Furthermore, our results revealed that MYB108 interacts with the promoter of *ANAC003*, while not with those of *ANAC010*, *ANAC042* or *ANAC075*, suggesting different control mechanisms involved in regulating ANAC010, ANAC042 and ANAC075 expression. Earlier studies have shown WRKY, bZIPs, HB TFs, or other NAC proteins could be other possible upstream regulatory factors [32]. Taken together, our results suggest that MYB-NAC TFs regulatory singling is involved in the dark induced Tic55-associated plant senescence by a complex transcriptional network.

## 3. Materials and Methods

### 3.1. Plant Materials and Growth Conditions

All *A. thaliana* plants used in this study were of the Columbia ecotype. *tic40-2* mutant plants were isolated by screening T2 seeds from a T-DNA insertion population for pale-green phenotypes (mutant line 2490) and the T-DNA insertion position was identified by plasmid rescue [79]. *A. thaliana* plants were grown as described previously [80]. For in vitro growth, seeds were surface sterilized, sown on 1/2× MS agar containing 2% (*w*/*v*) sucrose in petri dishes, and kept in a growth chamber (22 °C, 16-h light/8-h dark). To select for the T-DNA insertion mutants, kanamycin monosulfate was added to the MS medium at a final concentration of 50 µg/mL. Seedlings of *A. thaliana* plants were grown under a long-day cycle (22 °C, 16-h light/8-h dark).

### 3.2. Preparation of Chloroplasts and Chloroplast Subfractions

Intact chloroplasts were isolated from 20-day-old *A. thaliana* plants, as described previously [81,82]. Outer membrane (OM), inner membrane (IM), thylakoid, and stromal fractions were recovered and isolated from the supernatant through sucrose density centrifugation [83].

### 3.3. Protein Gel Blot Analysis

Proteins were separated through SDS-PAGE by using SDS gels (NuPAGE 4–12% Bis-Tris Gel; Invitrogen, Carlsbad, CA, USA) and electroblotted on to PVDF membrane (Immobulilin P; Millipore, Bedford, MA, USA), according to the standard procedures. The membranes were immunodetected using the method of Harlow and Lane [84], with an alkaline phosphatase-linked second antibody and bromochloroindoyl phosphate (BCIP)/nitro blue tetrazolium (NBT) development.

### 3.4. Coimmunoprecipitation (Co-IP) Analysis

Intact chloroplasts were isolated from 20-day-old *A. thaliana* plants, as has been described in previous research [81,82]. Briefly, 20-day-old *A. thaliana* plants were ground in 1× grinding buffer (GB; 330 mM D-sorbitol, 50 mM HEPES-KOH, pH 8.0, 2 mM EDTA, 0.5% BSA) and filtered through two layers of Miracloth (Calbiochem, Inc., La Jolla, CA, USA). After centrifugation, the pellet was dissolved in 1× GB buffer. The chloroplast was then isolated and resuspended in 1× import buffer (600 mM D-sorbitol, 100 mM HEPES-KOH, pH 8.0) to a final concentration of 1 mg/mL after Percoll (Amersham Biosciences, UPpsala, Sweden) gradient centrifugation. Diluted chloroplast (0.5 mg/mL) was then treated with 0.5 mM dithiobis succinimidylpropionate (DSP) and 50 mM glycine, followed by 2× hypotonic buffer (25 mM HEPES-KOH, pH 8.0, 4 mM MgCl_2_). After ultracentrifugation at 45,000 rpm (Beckman TL-100), the pellet was dissolved in 1× solubilization buffer (25 mM HEPES-KOH, pH 8.0, 50 mM KCl, 4% Tx-100, 20% glycerol, proteinase inhibitor) and incubated on ice for 10 min. Subsequently, 50 μL of supernatant was collected, mixed with 2× sample buffer (900 mM Tris-HCl, pH 8.45, 24% glycerol, 8% SDS, 0.0076% Coomassie Blue G, 0.01% Phenol Red, 0.001 mM EDTA, 0.1 mM DTT), and stored at −20 °C for further study after centrifugation. The leftover supernatant was diluted with HKG buffer (25 mM HEPES-KOH, pH 8.0, 50 mM KCl, 10% glycerol) and divided into two tubes. Anti-Tic55 antibody and preimmune antibody were added into separate tubes; protein A was then added into each tube following 12 h incubation at 4 °C. After 4 h, the pellet was washed four times with equal volume of 2× solubilization buffer and 2× HKG buffer after centrifugation. Finally, the pellet was resuspended in 30 μL of 2× sample buffer and protein gel blot was performed with specific antibodies.

### 3.5. Dark Treatment and Chlorophyll Concentration Analysis

Dark-induced aging: Following Wada and others [85], the third to sixth leaves of 19-day-old WT (Columbia ecotype) and mutant (*tic55-II*-knockout) *A. thaliana* plants were either covered with aluminum foil to prevent exposure to sunlight or left untreated and collected directly as untreated controls (Day 0). On Day 5, the IDLs were uncovered and both they and the untreated (control) leaves were photographed for both WT and *tic55-II*-knockout plants

Chlorophyll concentration analysis: Quantitative analysis of chlorophyll a/b concentration was performed according to the method described by Porra [86]. Briefly, we collected the fifth and sixth leaves from normal and dark-treated 19-day-old seedlings; these were weighed, frozen in liquid nitrogen, and finally stored at −80 °C. The frozen leaves were then broken in 80% acetone and centrifuged. Chlorophyll was extracted and collected from the supernatant by repeating the preceding steps until the centrifuged pellets were almost completely white. The absorbance of the collected chlorophyll at 663 and 647 nm (A663 and A647, respectively) was determined; chlorophyll concentrations were then calculated as follows [87]:

Chlorophyll a (µg/mL) = (12.25 × A663) − (2.55 × A647)

Chlorophyll b (µg/mL) = (20.31 × A647) − (4.91 × A663)

Total chlorophyll (µg/mL) = (18.71 × A647) + (7.15 × A663).

Finally, the chlorophyll concentrations were divided by the weight of the leaf tissues and multiplied by the total collected volume.

### 3.6. Hybridization of A. thaliana Microarrays and Data Analysis

Total RNA (0.2 μg), isolated from each sample by using TRIzol (Invitrogen) according to the manufacturer’s instructions, was amplified by using Low Input Quick-Amp Labeling Kit (Agilent Technologies, Santa Clara, CA, USA) and labeled with Cy3 (CyDye, Agilent Technologies) during the in vitro transcription process. Cy3-labeled cRNA (1.65 μg) was fragmented to an average size of approximately 50–100 nucleotides through incubation with fragmentation buffer at 60 °C for 30 min. Correspondingly fragmented and labeled cRNA was then pooled and hybridized using a Agilent Arabidopsis V4 Oligo 4 × 44 K Microarray (Agilent Technologies) at 65 °C for 17 h. After being washed and dried through nitrogen gun blowing, the hybridized microarrays were scanned on an Agilent microarray scanner (Agilent Technologies) at 535 nm for Cy3. The scanned images were analyzed using Feature extraction 10.5.1.1 software (Agilent Technologies); image analysis and normalization software were used to quantify signal and background intensity for each feature, respectively. The data points with nonzero flag values or <2.6 signal-to-noise ratios were masked. The remaining data were log_2_-transformed and averaged for each gene. For selecting genes significantly expressed in the *tic55-II*-knockout mutant after dark treatment compared with the WT, a 2-fold change and *p*ValueLogRatio of <0.05 were used as thresholds. The GO Slim Classification for Plants, developed at TAIR (http://www.arabidopsis.org/help/helppages/go_slim_help.jsp), was used to characterize the functionally upregulated and downregulated genes. The GO identifier of the optimal hit (with a cutoff of *E*-value < 10^−5^) was attributed to the sequence. This step allowed putative functions to be assigned on the basis of the classification proposed by the GO.

### 3.7. Relative Real-Time qRT-PCR and Microarray Validation

Real-time qRT-PCR was used to validate selected data from the microarray experiments and examine the expression of a subset of genes over time. The leaf senescence-related genes, namely *ASP3*, *APG7, DIN2*, *DIN11*, *SAG12*, *SAG13* and *YLS9*, and primers used for the qRT-PCR assays are listed in Table S1. The cDNA was synthesized from 1 µg of total RNA in a volume of 20 μL using the GoScript^TM^ Reverse Transcription System (Promega, Madison, WI, USA), according to the manufacturer’s instructions. The cDNA products were diluted and used for real-time qPCR analysis, which was performed using a PTC-200 thermal cycler and real-time fluorescence monitoring by a Chromo 4 optical detector (MJ Research/Bio-Rad, Hercules, CA, USA) with the SYBR Green Master Mix (Toyobo) for transcript measurements. Amplification conditions were as follows: one cycle at 95 °C for 1 min, followed by 35 cycles of 95 °C for 15 s and then 60 °C for 60 s, with plate reading conducted after each cycle. The *Arabidopsis* tubulin gene (*TUB2*) was used as the endogenous control. After the completion of PCR amplification, all data from three replicated qRT-PCR samples were analyzed using Bio-Rad CFX Manager^TM^ (version 1.5.534.0511, Hercules, CA, USA) to the 2^−ΔΔ*C*t^ method [87].

### 3.8. Transactivation Assays in Yeast Cells

A yeast one-hybrid assay (Clontech) was performed to determine whether MYB108 can activate the promoter regions of downstream target transcription factors, including ANAC003, ANAC010, ANAC042 and ANAC075, which were chosen based on the results of our microarray analysis in *Arabidopsis*. The MYB108 ORF was amplified using PCR reaction incorporating *A. thaliana* cDNA as a template. The products were cloned into pDONR221 by Gateway BP clonase II Enzyme Mix (Invitrogen), subsequently fused to the GAL4 activation domain (AD) in the pGADT7 vector to create pGADT7-MYB108 by Gateway LR clonase II Enzyme Mix (Invitrogen). Around 1 Kb DNA fragments located upstream of the predicted transcription start site of *ANAC003*, *ANAC010*, *ANAC042* and *ANAC075*, respectively contain potential sequences binding to MYB. These promoter regions were amplified by PCR reactions using primers containing *SacI* and *SpeI* restriction sites and then cloned into the pHIS2.1 vector (Clontech) to generate recombinant constructs: pHIS2.1-*ANAC003p*, pHIS2.1-*ANAC010p*, pHIS2.1-*ANAC042p* and pHIS2.1-*ANAC075p*. Thereafter, pGADT7-MYB108 and pHIS2.1-*ANAC003p* (or pHIS2.1-*ANAC010p,* or pHIS2.1-*ANAC042p,* or pHIS2.1-*ANAC075p*) were co-transformed into yeast AH109 strain, followed by grown for three days on SD/–Leu/–Trp medium and SD/–Leu/–Trp/–His medium with or without 5 mM 3-AT to investigate the expression of the reporter gene *HIS3*. Plasmid pGADT7 alone was used as a negative control.

## 4. Conclusions

Tic55 is associated with several translocon proteins (such as Tic32, Tic62, Tic110, and Tic40) located at the IM of chloroplasts in peas, and functions as a chloroplast protein importer [18,19,20]. Of note, Tic55 executes its activity as a hydroxylase of phyllobilins during plant senescence in *A. thaliana* [22], however, the regulatory network remains unclear. The characterization of a *tic55-II*-knockout mutant, which generated neither *tic55* mRNA nor protein, showed no significant phenotypic differences between WT and *tic55-II*-knockout *A. thaliana*, suggesting that Tic55 is not functionally essential for its survival. A unique biological function of Tic55 was finally revealed when *A. thaliana* was aged under dark treatment and the senescent leaves were analyzed using microarray technology. Based on microarray analysis results, seven senescence-associated genes (SAGs) were selected for qRT-PCR analysis. The results of qRT-PCR analysis correlated closely with those of microarray analysis, indicating the reliability of the microarray results (Figure 7). The absence of Tic55 in the *tic55-II*-knockout mutant indirectly inhibited the expression of these seven senescence-related genes, subsequently delaying leaf senescence. Our data thus indicate that the novel biological function of Tic55 is related to the dark-induced aging of *A. thaliana*. This is supported by the results of Hauenstein et al. [22], who indicated that Tic55 functions as a hydroxylase and is involved in chlorophyll breakdown during plant senescence. However, their work mainly focuses on the elucidation of the biological function of Tic55, whereas our study reveals not only the direct effect of Tic55 on chlorophyll catabolism but also its indirect role in the downstream senescence associated genes (*SAGs*) expression through microarray and qRT-PCR analyses. Furthermore, yeast one-hybrid assays confirmed the expression of the NAC gene, *ANAC003*, was likely controlled by MYB108, which was also downregulated in dark stressed *tic55-II*-knockout mutant. Our studies thus shed light on further understanding the biological function of chloroplast protein Tic55 and its association with MYB-NAC network signaling involved in the dark-induced aging, since researches regarding light-related senescence are relatively rare.

## Figures and Tables

**Table 1:** List of significantly upregulated or downregulated genes in aging, senescence, and leaf senescence subcategories from biological processes categories according to the gene ontology classification scheme from microarray analysis. Data provided represent fold expression Log_2_ (*tic55-II*-knockout/wild type).

Locus ID	GO Term	Annotation	Up or Down	Fold Change (*tic55-II*/WT)
NM_180632	GO:0007568~aging GO:0010149~senescence GO:0010150~leaf senescence	*Arabidopsis thaliana* epithiospecifier protein mRNA (ESP)	Up	2.452
NM_124040	GO:0007568~aging	*Arabidopsis thaliana* tetraspanin family protein mRNA (TRN2)	Up	2.017
NM_001036860	GO:0007568~aging GO:0010149~senescence	*Arabidopsis thaliana* vegetative storage protein 2 mRNA (VSP2)	Up	2.009
NM_121190	GO:0007568~aging GO:0010149~senescence GO:0010150~leaf senescence	*Arabidopsis thaliana* aspartate 3 aminotransferase mRNA (ASP3)	Down	−2.107
NM_129157	GO:0007568~aging GO:0010149~senescence GO:0010150~leaf senescence	*Arabidopsis thaliana* late embryogenesis abundant hydroxyproline-rich glycoprotein mRNA (YLS9)	Down	−2.52
NM_123958	GO:0007568~aging GO:0010149~senescence GO:0010150~leaf senescence	*Arabidopsis thaliana* ubiquitin-like modifier-activating enzyme atg7 mRNA (APG7)	Down	−2.533
NM_201829	GO:0007568~aging	*Arabidopsis thaliana* senescence-associated protein 13 mRNA (SAG13)	Down	−3.353
NM_115877	GO:0007568~aging	*Arabidopsis thaliana* beta-glucosidase 30 mRNA (DIN2)	Down	−3.735
NM_115877	GO:0007568~aging	*Arabidopsis thaliana* 2-oxoacid-dependent dioxygenase-like protein DIN11 mRNA (DIN11)	Down	−3.735
NM_123957	GO:0007568~aging GO:0010149~senescence GO:0010150~leaf senescence	*Arabidopsis thaliana* cysteine protease mRNA (SAG12)	Down	−7.388

**Table 2:** Possible MYB binding sites predicted by PlantPAN 2.0 analyses [78].

NAC Genes	MYB Binding Sequences (Nucleotides No.) ^1^
*ANAC003*	AGAATATAGT (-222~-231); ATCGTATCTATGT (-329~-341); AGATAACGGA (-356~-365); AAAGATATGTC (-537~-547); TTGATATTT (-614~-622); GGAATATTTT (-658~-667)
*ANAC010*	TTGGTAGGTC (-33~-42); AAAATATTAT (-191~-200); TATAATCTGTT (-641~-651); TGAGATCTCT (-786~-795); GTGAAGATATGGT (-850~-862)
*ANAC042*	AGTTATCCTTT (-133~-143); TGTGAATCTTA (-152~-162); GATAATCTGA (-302~-311); TCAGATTCTCT (-643~-653); TAAGATCTTG (-845~-854)
*ANAC075*	AAGATATTCG (-9~-18); TTCATATCTTCAC (-132~-144); CGGTTAGGT (-218~-0226); TAAGATCTTA (-310~-319); TAAATATTTT (-725~-734); TTCATATCTC (-879~-888); TCTGATATTAT (-903~-913); ATAAAGATACATA (-932~-944)

^1^ Nucleotide sequence numbers correspond to the sequences upstream of the transcriptional start site of their respective NAC genes.

## Supplementary Materials

Supplementary materials can be found at http://www.mdpi.com/1422-0067/19/7/1854/s1.

## Abbreviations

Toc and Tic	Translocon at the outer/inner membrane of chloroplasts
IDLs	individually darkened leaves
*ASP3*	*aspartate aminotransferase 3*
*APG7*	*autophagy-related 7*, ubiquitin-like modifier-activating enzyme ATG7
*DIN2*	*dark inducible 2*, beta-glucosidase 30
*DIN11*	*dark inducible 11*,2-oxoacid-dependent dioxygenase-like protein
*SAG12*	*senescence-associated gene 12*, cysteine protease
*SAG13*	*senescence-associated gene 13*, senescence-associated protein
*YLS9*	*yellow-leaf-specific gene 9*, protein NDR1/HIN1-like 10
ABRC	Arabidopsis Biological Resource Center
NBT	nitro blue tetrazolium
BCIP	bromochloroindoyl phosphate
