# Supplementary material for: The Direct Involvement of Dark-Induced Tic55 Protein in Chlorophyll Catabolism and Its Indirect Role in the MYB108-NAC Signaling Pathway during Leaf Senescence in Arabidopsis thaliana
Source: Int J Mol Sci. 2018 Jun 23;19(7):1854. doi: 10.3390/ijms19071854 (PMC6073118; doi:10.3390/ijms19071854)
Supplement: Supplementary file 1 [file ijms-19-01854-s001.pdf]

International Journal of *Molecular Sciences*

MDPI

# Supplementary Materials: Dark-induced senescence protein Tic55 involved in MYB108 linked ANAC signaling pathway in *Arabidopsis thaliana*

**Ming-Lun Chou, Wan-Yu Liao, Wan-Chen Wei, Althea Yi-Shan Li, Ching-Ying Chu, Chia-Ling Wu, Chun-Lin Liu, Ting-Han Fu, and Lee-Fong Lin**

**Table S1.** Primers used in this study.

| Target genes | Primer name | Oligo sequence (5′ to 3′)[1] | Purpose |
|---|---|---|---|
| *atTic55* | atTic55-RT-F | GAGCATGCATAGTCCCAATTCCGCCTT | Semi qRT-PCR forward primer |
| | atTic55-RT-R | GAGAATCTGATGGATCCTGCTCATGTT | Semi qRT-PCR reverse primer |
| *atActin 2* | ACT2-RT-F | ACATTGCAAAGAGTTTCAAGGT | Semi qRT-PCR forward primer |
| | ACT2-RT-R | CTAAGCTCTCAAGATCAAAGG | Semi qRT-PCR reverse primer |
| peaTic 55 | psTic55S-F-NcoI | CATGCCATGGAGTTGGGTCTCAAAGTATA TGAT | yeast two-hybrid analysis, Tic55S PCR forward primer |
| | psTic55S-R-XhoI | CCGCTCGAG TCACAATCTCCTATGTACCCT | yeast two-hybrid analysis, Tic55S PCR reverse primer |
| peaTic 40 | psTic40S-F1-NcoI | CATGCCATGGAGCAGATGAATACACAAA ATAAT | yeast two-hybrid |

| | | | |
|---|---|---|---|
| | | | analysis, Tic40S/ Tic40TPR PCR forward primer |
| | psTic40S-F2-NcoI | CATGCCATGGAGGGGCTTTCTCCACAAGAAGTC | yeast two-hybrid analysis, Tic40Hip/Hop PCR forward primer |
| | psTic40S-R1-PstI | AACTGCAGTCAAGGGGGGCCTGAAACCCC | yeast two-hybrid analysis, Tic40S/ Tic40Hip/Hop PCR reverse primer |
| | psTic40S-R2-PstI | AACTGCAGAATTTGATCAAATTGTTGTTT | yeast two-hybrid analysis, Tic40TPR PCR reverse primer |
| peaTic 110 | psTic110S-F1-NcoI | CATGCCATGGAGAATGCTGCTGCACCGCAAGTT | yeast two-hybrid analysis, Tic110S/ Tic110N PCR forward primer |
| | psTic110S-F2-NcoI | CATGCCATGGAGCTCATCTCATTTAAGAACCAT | yeast two-hybrid analysis, Tic110C PCR forward primer |
| | psTic110S-R1-PstI | AACTGCAGCTAGAATACAAACTTCTCTTC | yeast two-hybrid analysis, Tic110S/ |

| | | | |
|---|---|---|---|
| | psTic110S-R2-PstI | AACTGCAGTAAATCATTAAATGCCAATAC | Tic110C PCR reverse primer yeast two-hybrid analysis, Tic110N PCR reverse primer |
| *APG7* | APG7-qRT-F | CTGGTTTAACCGAGCTTAAGAA | Real-time qRT-PCR forward primer |
| | APG7-qRT-R | CATCGTCTTCCCAGTCGAGGTT | Real-time qRT-PCR reverse primer |
| *ASP3* | ASP3-qRT-F | AGCTCAAGTCTCCTTCATGACT | Real-time qRT-PCR forward primer |
| | ASP3-qRT-R | ATCCTCCCGTCAGATGTCATGT | Real-time qRT-PCR reverse primer |
| *DIN11* | DIN11-qRT-F | TGCCAAAAGAGTCGTCTATGGA | Real-time qRT-PCR forward primer |
| | DIN11-qRT-R | TTTGCAAAGGTTGTCAGGACTT | Real-time qRT-PCR reverse primer |
| *DIN2* | DIN2-qRT-F | CGTTGCTCCTTTGATTTTCCTC | Real-time qRT-PCR forward primer |
| | DIN2-qRT-R | TGGGTTCTCTATGCCTTGCAAA | Real-time qRT-PCR reverse primer |
| *SAG12* | SAG12-qRT-F | TGCAGTAACTGCGATTGGATAC | Real-time qRT-PCR |

| | | | |
|---|---|---|---|
| | | | forward primer |
| | SAG12-qRT-R | TTGATGATCCAATACTTTGATC | Real-time qRT-PCR reverse primer |
| *SAG13* | SAG13-qRT-F | ATTTAGATGTGTCCACATGTTC | Real-time qRT-PCR forward primer |
| | SAG13-qRT-R | CCACGCAAGCATAAATATCTAA | Real-time qRT-PCR reverse primer |
| *YLS9* | YLS9-qRT-F | TTTAAGTACCTCAAACGGAACT | Real-time qRT-PCR forward primer |
| | YLS9-qRT-R | AAGTCGCACTTGATCGGGAAAA | Real-time qRT-PCR reverse primer |
| *ANAC 003* | AtNAC003-qRT-F | TGATACCGCCGAGAAGAAGAA | Real-time qRT-PCR forward primer |
| | AtNAC003-qRT-R | TGACCCAATTTATGCTCACCA | Real-time qRT-PCR reverse primer |
| *ANAC 010* | AtNAC010-qRT-F | CGGGTATGAGCATGGTGGTAA | Real-time qRT-PCR forward primer |
| | AtNAC010-qRT-R | TCGCCTTCACGAACTACCAA | Real-time qRT-PCR reverse primer |
| *ANAC 042* | AtNAC042-qRT-F | TAGATTCCGACCACACGAGC | Real-time qRT-PCR forward primer |
| | AtNAC042-qRT-R | ATTCTGTGGCTGTGGAAGCG | Real-time |

| | | | |
|---|---|---|---|
| | | | qRT-PCR reverse primer |
| *ANAC 075* | AtNAC075-qRT-F | ATGGGGAACCAACAAGAAGCA | Real-time qRT-PCR forward primer |
| | AtNAC075-qRT-R | TGTCAGAAGAGTCAGGGGCT | Real-time qRT-PCR reverse primer |
| *Tubulin 2* | TUB2/qRT-F | TGGCATCAACTTTCATTGGA | Real-time qRT-PCR forward primer |
| | TUB2/qRT-R | ATGTTGCTCTCCGCTTCTGT | Real-time qRT-PCR reverse primer |
| *ANAC 003* promoter | AtNAC003p-F-SacI | TCGAGCTCGCCAAAATAATAAAATCGCGT | yeast one-hybrid analysis, genomic DNA PCR forward primer |
| | AtNAC003p-R-SpeI | GGACTAGTCTCTGCAGCGATCAAAAAAGA | yeast one-hybrid analysis, genomic DNA PCR reverse primer |
| *ANAC 010* promoter | AtNAC010p-F-SacI | TCGAGCTCGGTCTACTTTAAGACACAGAT | yeast one-hybrid analysis, genomic DNA PCR forward primer |
| | AtNAC010p-R-SpeI | GGACTAGTCCTTCTTATTTTACTGATCTC | yeast one-hybrid analysis, |

| | | | |
|---|---|---|---|
| | | | genomic DNA PCR reverse primer |
| *ANAC 042* promoter | AtNAC042p-F-SacI | TCGAGCTCGGAATGCAGTTGAAGGAATTG | yeast one-hybrid analysis, genomic DNA PCR forward primer |
| | AtNAC042p-R-SpeI | GGACTAGTCTTCACTACATCGATCTCTTT | yeast one-hybrid analysis, genomic DNA PCR reverse primer |
| *ANAC 075* promoter | AtNAC075p-F-SacI | TCGAGCTCGTTAATAGCGATGAAAATTTC | yeast one-hybrid analysis, genomic DNA PCR forward primer |
| | AtNAC075p-R-SpeI | GGACTAGTCTCAATCTCGAATATCTTTGA | yeast one-hybrid analysis, genomic DNA PCR reverse primer |
| *MYB 108* | AtMYB108-F-NcoI | CATGCCATGGATGAAAAAGGAAGAAGCTTGAAG | yeast one-hybrid analysis, RT-PCR forward primer |
| | AtMYB108-R-ClaI | CCATCGATCAGAAGCTACCATTATTGTTGAACTG | yeast one-hybrid analysis, RT-PCR reverse |

primer

[1] Underlined sequences represent respective restriction enzyme sites.

**Table S2**. Number of genes that upregulated or downregulated (2-fold differentially expressed transcripts) according to the GO classification scheme in the dark-induced leaf senescence identified by microarray analysis.

| **Category** | **GO Term** | | **No. of upregulated transcripts (Positive values)** | **No. of downregulated transcripts (Negative values)** |
|---|---|---|---|---|
| Biological_process | GO:0007568 | aging | 3 | -7 |
| | GO:0009072 | aromatic amino acid family metabolic process | 0 | -5 |
| | GO:0019438 | aromatic compound biosynthetic process | 0 | -12 |
| | GO:0009851 | auxin biosynthetic process | 0 | -3 |
| | GO:0010120 | camalexin biosynthetic process | 0 | -3 |
| | GO:0052317 | camalexin metabolic process | 0 | -3 |
| | GO:0046394 | carboxylic acid biosynthetic process | 5 | -11 |
| | GO:0009830 | cell wall modification during abscission | 0 | -2 |
| | GO:0022411 | cellular component disassembly | 0 | -3 |
| | GO:0070301 | cellular response to hydrogen peroxide | 2 | -5 |
| | GO:0034599 | cellular response to oxidative stress | 2 | -6 |
| | GO:0034614 | cellular response to reactive oxygen species | 2 | -6 |
| | GO:0033554 | cellular response to stress | 5 | -14 |
| | GO:0006825 | copper ion transport | 1 | -3 |
| | GO:0006952 | defense response | 13 | -30 |
| | GO:0042742 | defense response to bacterium | 4 | -7 |
| | GO:0009814 | defense response, incompatible interaction | 1 | -7 |
| | GO:0042744 | hydrogen peroxide catabolic process | 2 | -5 |
| | GO:0042743 | hydrogen peroxide metabolic process | 2 | -5 |
| | GO:0006955 | immune response | 1 | -12 |
| | GO:0046219 | indolalkylamine biosynthetic process | 0 | -3 |
| | GO:0006586 | indolalkylamine metabolic process | 0 | -4 |

| | | | | |
|---|---|---|---|---|
| | GO:0042430 | indole and derivative metabolic process | 0 | -6 |
| | GO:0042435 | indole derivative biosynthetic process | 0 | -6 |
| | GO:0042434 | indole derivative metabolic process | 0 | -6 |
| | GO:0042431 | indole metabolic process | 0 | -3 |
| | GO:0009700 | indole phytoalexin biosynthetic process | 0 | -3 |
| | GO:0046217 | indole phytoalexin metabolic process | 0 | -3 |
| | GO:0045087 | innate immune response | 1 | -11 |
| | GO:0010386 | lateral root primordium development | 0 | -3 |
| | GO:0010150 | leaf senescence | 1 | -4 |
| | GO:0009809 | lignin biosynthetic process | 0 | -4 |
| | GO:0016042 | lipid catabolic process | 5 | -8 |
| | GO:0010876 | lipid localization | 7 | -3 |
| | GO:0006869 | lipid transport | 7 | -3 |
| | GO:0030001 | metal ion transport | 2 | -9 |
| | GO:0009556 | microsporogenesis | 1 | -3 |
| | GO:0010260 | organ senescence | 1 | -4 |
| | GO:0016053 | organic acid biosynthetic process | 5 | -11 |
| | GO:0055114 | oxidation reduction | 9 | -41 |
| | GO:0006800 | oxygen and reactive oxygen species metabolic process | 2 | -6 |
| | GO:0052315 | phytoalexin biosynthetic process | 0 | -3 |
| | GO:0052314 | phytoalexin metabolic process | 0 | -3 |
| | GO:0048236 | plant-type spore development | 1 | -3 |
| | GO:0010817 | regulation of hormone levels | 2 | -6 |
| | GO:0009733 | response to auxin stimulus | 13 | -11 |
| | GO:0009617 | response to bacterium | 6 | -12 |
| | GO:0009719 | response to endogenous stimulus | 23 | -38 |
| | GO:0009723 | response to ethylene stimulus | 3 | -9 |
| | GO:0009725 | response to hormone stimulus | 20 | -30 |
| | GO:0042542 | response to hydrogen peroxide | 2 | -5 |
| | GO:0009753 | response to jasmonic acid stimulus | 4 | -12 |
| | GO:0010033 | response to organic substance | 24 | -46 |
| | GO:0006979 | response to oxidative stress | 3 | -11 |
| | GO:0010193 | response to ozone | 0 | -4 |
| | GO:0000302 | response to reactive oxygen species | 2 | -7 |
| | GO:0009751 | response to salicylic acid stimulus | 2 | -7 |
| | GO:0009636 | response to toxin | 1 | -3 |
| | GO:0009611 | response to wounding | 2 | -13 |

| | | | | |
|---|---|---|---|---|
| | GO:0048364 | root development | 3 | -8 |
| | GO:0022622 | root system development | 3 | -8 |
| | GO:0009863 | salicylic acid mediated signaling pathway | 1 | -3 |
| | GO:0019748 | secondary metabolic process | 1 | -16 |
| | GO:0010149 | senescence | 2 | -4 |
| | GO:0000041 | transition metal ion transport | 2 | -4 |
| | GO:0000162 | tryptophan biosynthetic process | 0 | -3 |
| | GO:0006568 | tryptophan metabolic process | 0 | -4 |
| Cellular_component | GO:0048046 | apoplast | 11 | -8 |
| | GO:0005618 | cell wall | 14 | -18 |
| | GO:0012505 | endomembrane system | 37 | -99 |
| | GO:0030312 | external encapsulating structure | 14 | -19 |
| | GO:0005576 | extracellular region | 23 | -27 |
| | GO:0044421 | extracellular region part | 0 | -4 |
| | GO:0009505 | plant-type cell wall | 5 | -8 |
| Molecular_function | GO:0003993 | acid phosphatase activity | 2 | -2 |
| | GO:0004022 | alcohol dehydrogenase (NAD) activity | 0 | -3 |
| | GO:0005275 | amine transmembrane transporter activity | 1 | -4 |
| | GO:0016209 | antioxidant activity | 2 | -7 |
| | GO:0004190 | aspartic-type endopeptidase activity | 2 | -4 |
| | GO:0070001 | aspartic-type peptidase activity | 2 | -4 |
| | GO:0005509 | calcium ion binding | 5 | -11 |
| | GO:0004091 | carboxylesterase activity | 5 | -14 |
| | GO:0043169 | cation binding | 22 | -96 |
| | GO:0045551 | cinnamyl-alcohol dehydrogenase activity | 0 | -3 |
| | GO:0048037 | cofactor binding | 1 | -18 |
| | GO:0015082 | di-, tri-valent inorganic cation transmembrane transporter activity | 1 | -4 |
| | GO:0019104 | DNA N-glycosylase activity | 2 | -1 |
| | GO:0009055 | electron carrier activity | 4 | -34 |
| | GO:0004175 | endopeptidase activity | 3 | -10 |
| | GO:0004857 | enzyme inhibitor activity | 1 | -9 |
| | GO:0050660 | FAD binding | 1 | -10 |
| | GO:0046527 | glucosyltransferase activity | 0 | -8 |
| | GO:0020037 | heme binding | 3 | -22 |
| | GO:0016799 | hydrolase activity, hydrolyzing | 2 | -1 |

| | | | |
|---|---|---|---|
| | N-glycosyl compounds | | |
| GO:0043167 | ion binding | 22 | -96 |
| GO:0005506 | iron ion binding | 5 | -32 |
| GO:0008289 | lipid binding | 7 | -6 |
| GO:0016165 | lipoxygenase activity | 0 | -3 |
| GO:0046872 | metal ion binding | 18 | -86 |
| GO:0045735 | nutrient reservoir activity | 4 | -3 |
| GO:0016717 | oxidoreductase activity, acting on paired donors, with oxidation of a pair of donors resulting in the reduction of molecular oxygen to two molecules of water | 2 | -1 |
| GO:0016684 | oxidoreductase activity, acting on peroxide as acceptor | 2 | -6 |
| GO:0016701 | oxidoreductase activity, acting on single donors with incorporation of molecular oxygen | 0 | -5 |
| GO:0019825 | oxygen binding | 1 | -15 |
| GO:0046910 | pectinesterase inhibitor activity | 0 | -6 |
| GO:0004601 | peroxidase activity | 2 | -6 |
| GO:0080043 | quercetin 3-O-glucosyltransferase activity | 0 | -4 |
| GO:0080044 | quercetin 7-O-glucosyltransferase activity | 0 | -3 |
| GO:0046906 | tetrapyrrole binding | 6 | -22 |
| GO:0046914 | transition metal ion binding | 12 | -71 |
| GO:0035251 | UDP-glucosyltransferase activity | 0 | -8 |

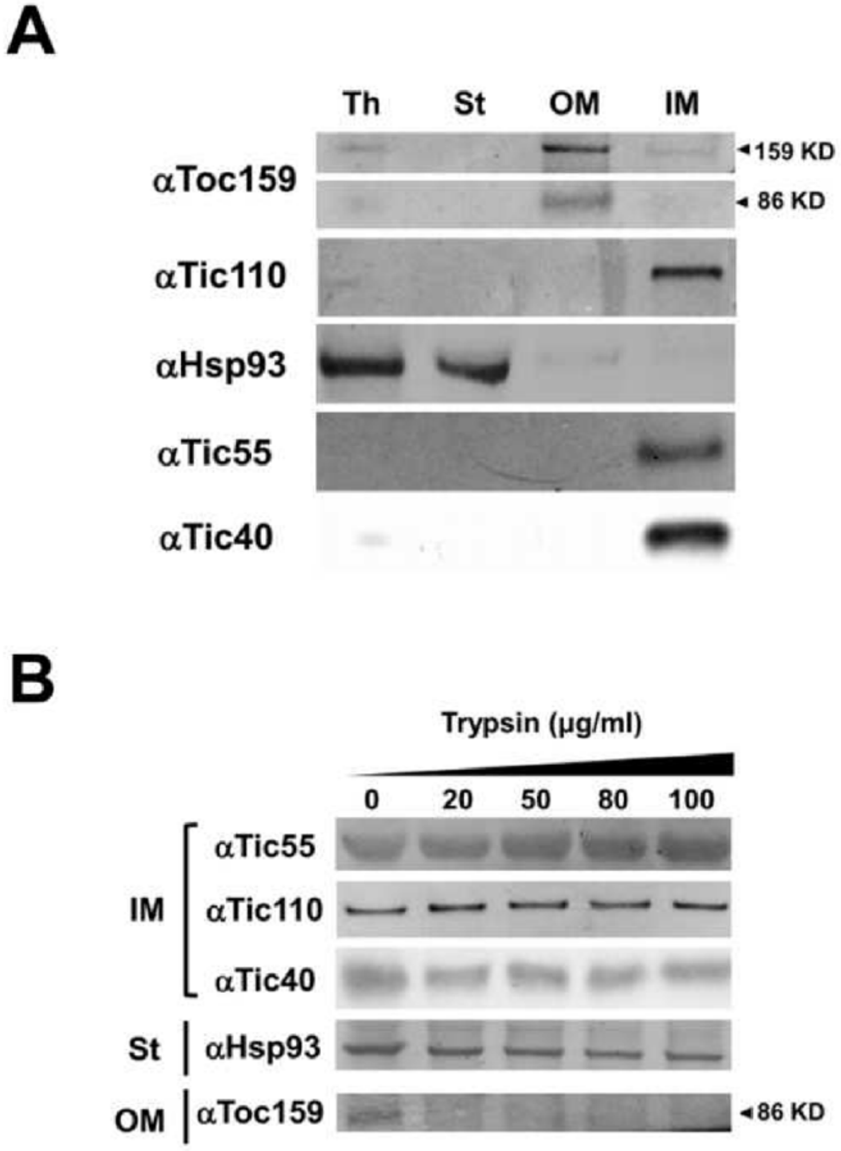


**Figure S1.** Tic55 situates at the inner membrane of chloroplast. (**A**) Fractionation analysis of chloroplast was carried out as described in the Materials and Methods section. Thylakoid proteins (Th), Stoma proteins (St), Outer membrane proteins (OM), and Inner membrane proteins (IM) were successfully separated; protein gel blots were performed using specific antibodies against the translocon proteins Toc159, Tic110, Hsp93, Tic55, and Tic40, respectively. (**B**) Protease Trypsin treatment to determine the protein orientation of Tic55 at the inner membrane of chloroplast. Isolated chloroplasts were treated with different concentrations of trypsin (0 μg/mL-100 μg/mL), followed by protein gel blot analysis with specific antibodies. IM: inner membrane. St: stroma. OM: outer membrane.
